# A secondary analysis of the factors associated with women´s adequate utilization of antenatal care services during their last pregnancy in Uganda

**DOI:** 10.1186/s12884-023-05994-8

**Published:** 2023-09-26

**Authors:** Moses Festo Towongo, Enock Ngome, Kannan Navaneetham, Gobopamang Letamo

**Affiliations:** Department of Population Studies, University of Botswana, Gaborone, Botswana

**Keywords:** Individual-level, Community-level, Adequate ANC services, Uganda

## Abstract

**Background:**

Adequate antenatal care services (ANC) use is critical to identifying and reducing pregnancy risks. Despite the importance placed on adequate antenatal care service utilization, women in Uganda continue to underutilize antenatal care services. The primary goal of this study is to identify the factors associated with women’s adequate utilization of antenatal care services in Uganda.

**Methods:**

Secondary data from the 2016 Uganda Demographic and Health Survey were used in this study. The study sample consists of 9,416 women aged 15 to 49 who reported giving birth in the five years preceding the survey. The adequate use of antenatal care services is the dependent variable. A woman who used antenatal care services at least four times is considered to have adequately used antenatal care services. We used univariate, bivariate, and multilevel logistic regression modelling to identify the factors associated with adequate utilization of antenatal care services. STATA version 14.2 was used to analyze the data.

**Results:**

The prevalence of adequate utilization of antenatal care services in Uganda was found to be 61.4%. Women with secondary or higher education were 32.0% (AOR = 1.32, 95% CI;1.07–1.63), employed women were 26.0% (AOR = 1.26, 95% CI;1.10–1.44), women who received high-quality antenatal care content were 78.0% (AOR = 1.78, 95% CI;1.58–2.02), and women who belong to the rich category of the wealth index bracket were 27.0% (AOR = 1.27, 95% CI;1.09–1.49), more likely to use antenatal care services adequately. Finally, the study discovered that women from less diverse ethnic communities were 15.0% (AOR, 0.85, 95%CI; 0.73–0.99) less likely to use antenatal care services adequately.

**Conclusion:**

Women’s adequate utilization of antenatal care was influenced by both community and individual-level characteristics. Policymakers must use a multi-sectoral approach to develop policies that address both individual and community-level characteristics.

## Introduction

Adequate utilization of antenatal care services (ANC) is imperative in identifying and reducing risks related to pregnancy [1, 2]. Adequate utilization of antenatal care services involves a woman utilizing antenatal care services at least four times during pregnancy based on the World Health Organization (WHO) focused ANC framework [3]. Following the adoption of the Sustainable Development Goals (SDGs), the WHO has equally modified its recommendation that a woman must visit ANC services at least four to eight times [4, 5].This is aimed at saving the lives of the mother and unborn baby as well as achieving the global agenda 3.1 goal of reducing maternal mortality to 70 deaths per 100,000 live births by 2030 [5]. Besides, adequate and early utilization of antenatal care services accords women with an opportunity for early screening for complications, referrals, treatment and also aids in continuous monitoring by skilled health personnel. It also aids in developing rapport between the woman and the service provider; during ANC contacts, women are educated on the danger signs of pregnancy, prevention and treatment, nutrition, breastfeeding, and contraceptives for family planning [6, 7]. Studies have shown that effective utilization of antenatal care services helps reduce maternal and neonatal mortality by 20% [8, 9]. Despite the significance placed on the adequate utilization of antenatal care services, in developing countries, ANC services are underutilized, and these ranged from 18.2% in Afghanistan to 92.2% in Jordan [10]. Moreover, in sub-Saharan Africa, studies have shown that adequate utilization of antenatal care services is low (6.8%), and majority of these countries still do not conform to the new WHO guidelines [11, 12]. Similarly, Studies have shown that Uganda is among the countries experiencing challenges in providing quality antenatal care services [13–16]. In 2003, Uganda adopted the WHO-focused antenatal care framework, which recommends at least four visits to ANC services for normal pregnancy. However, this was modified in 2018 to include eight contacts [17, 18]. Despite the adoption of WHO frameworks, ANC services in Uganda are underutilized [17]. Four repeated cross-sectional surveys using Uganda Demographic and Health Survey (UDHS) data (1995, 2000, 2006, and 2011) show that a higher proportion (over 90%) from 2001 to 2011 utilized at least one ANC service from skilled personnel, but less than half utilized ANC services adequately [19]. Besides, the recent UDHS 2016 results also showed that over half (60%) of the women visited antenatal care services at least four times, compared to 97% of the women who received at least one antenatal care from skilled health personnel [20]. This has kept maternal morbidity and mortality very high over time [20–22]. Globally, maternal mortality has receded by 38% [23]. However, maternal mortality remained unacceptably high in developing countries [24]. In 2020, an estimated 287 000 women died from maternal causes worldwide, which equates to about 800 maternal deaths per day, or one every two minutes [25]. The vast majority of these deaths were avoidable and occurred in developing countries. South Asia accounted for one-third of global maternal and neonatal mortality, while Sub-Saharan Africa accounted for more than two-thirds of these deaths [25]. In Uganda, the maternal mortality ratio is very high, estimated at 336 deaths per 100,000 live births [20]. The slow reduction of maternal mortality in Uganda continues to threaten the achievement of both the global and national agendas of reducing maternal mortality to 70 deaths and 15 deaths per 100,000 live births by 2030 and 2040, respectively [5, 26]. Several studies [19, 27–43] have identified factors that influenced the adequate utilization of antenatal care services. These factors include: regional variation [19, 27, 29–32], marital status [27, 36, 38], household wealth index [31–33, 36–38], level of education [27, 31, 32, 36, 38], distance to a health facility [27, 29, 37], cost [41, 42] media exposure [30, 37], maternal age [27, 31, 36–38], multigravida [31, 32, 37], mother’s occupation [31, 33, 36, 37], type of family [30], religion [27, 31, 34, 38], ethnicity [27, 29, 34, 36, 43], pregnancy wanted [29, 32, 37], sex of the household head [27, 31, 33], mean household size [30], mistimed pregnancy [36], spouse level of education [29, 31, 38], place of residence [29, 31, 32, 37, 38], ever had a pregnancy terminated [38], working status [27] type of pregnancy [36], and quality of the content of antenatal care services [39, 40]. Besides, the majority of studies conducted on the adequate utilization of ANC services in Uganda have focused mainly on individual-level factors [6, 17, 44–49]. However, factors that influence maternal healthcare service utilization in low and middle-income countries are multifactorial and necessitate integrated solutions based on a community and institutional perspective [50]. Empirical studies have identified community-level factors associated with adequate antenatal care service utilization. These include women’s place of residence, geographic region, literacy level in the community, ethnic diversity, socioeconomic status, and health facility [11, 12, 51–54]. However, because of differences in context, the majority of these community-level studies have produced inconsistent results [12, 51–53]. A study in Kenya also found that individual and contextual-level factors were the determinants of adequate utilization of maternal health care services [52]. Nonetheless, research on community-level factors that influence adequate utilization of antenatal care services is extremely limited in Uganda. In addition, studies that applied standard multilevel model analysis to the Demographic and Health Survey (DHS) dataset ignored the complex design nature of the dataset [54, 55]. Therefore, complex surveys such as the DHS that involve stratification typically use sampling, yielding selection probabilities that vary according to the stratum to which the population belongs. Therefore, ignoring the design aspect in standard multilevel modeling can lead to biased parameter estimates [56, 57]. Therefore, this study adopted Elkasabi et al. [58] revised methodology for multilevel analysis, which applies approximated level weights at individual and cluster-level to account for the complex nature of the data. This method helps to correct the problems of an inflated type one error and a large confidence interval. The findings from this study will provide evidence-based results which are generalizable to the entire Ugandan population. Further, the results from this study can be used by policy-makers and other stakeholders for effective policy-making and implementation.

## Data and methods

### Study sample

**Fig. 1:**
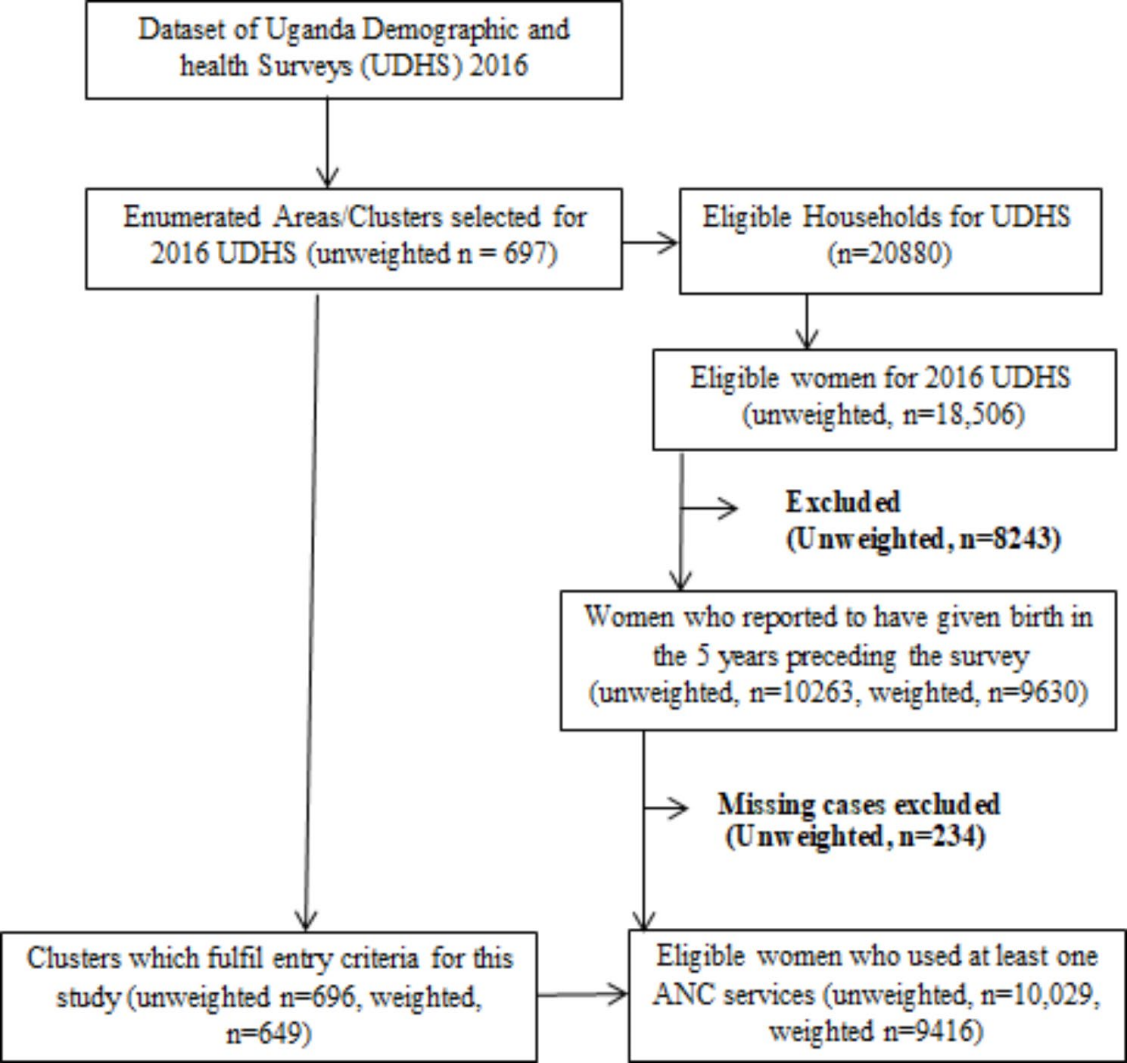
The data extraction procedure flowchart for UDHS 2016 in Uganda

Secondary data from the 2016 Uganda Demographic and Health Survey (UDHS) were used in this study. The 2016 UDHS used a two-stage stratified cluster sampling design with rural-urban and regional components. The Uganda Bureau of Statistics provided a sampling frame for the 2014 Uganda National Population and Housing Census (NPHC), which yielded a sample of 696 Enumerated Areas (EAs). The sample included a total of 20,791 households, with 19,938 of them occupied. A total of 19,588 occupied households were interviewed successfully, yielding a 98% response rate. The dataset was obtained with permission from the MEASURE International website (www.dhsprogram.com). A weighted sample of 9,416 women aged 15 to 49 who had given birth in the five years preceding the survey was included in this study. More information about the study design and sampling strategy can be found in the 2016 UDHS report [20] and the flowchart (See Fig. 1).

### Outcome variables

Adequate antenatal care services are the number of times a woman received ANC services; it was originally a numerical variable but was later classified as ‘four or more visits’ (coded as 1) and ‘less than four visits’ (coded as 0). A minimum of four visits to ANC services was considered because the WHO recommended it at the time of the survey [45]. The World Health Organization (WHO) recommends that a woman schedule her first visit within the first 12 weeks of her pregnancy, her second visit around week 26, her third visit around week 32, and her final visit between weeks 36 and 38 [3].

### Independent variables

Women’s education, employment status, place of residence, religion, parity, maternal age at last birth, marital status, household headship, family size, wealth index, and quality of antenatal care are all individual-level variables. Maternal age at last birth was calculated by subtracting the century month code (CMC) of the child’s birth date from the CMC of the mother’s birth date. Maternal age at last birth was divided into five categories: 15–19, 20–24, 25–29, 30–34, and 35+. Education is defined as the mother’s highest level of education and is classified as no education, primary, secondary, or higher. Depending on their response, women who reported working in the previous 12 months were classified as either employed or unemployed. The type of dwelling where the woman was discovered during the survey night was classified as rural or urban. Religion is defined as a woman’s religious affiliation and is classified as Anglican, Catholic, Muslim, and other religious groups. Parity is defined as the number of children a woman has had; it is classified as 1, 2–3, or 4 + children. Marital status is a legally defined marital state that is classified as single, married, living together, or previously married (separated, divorced, and widowed). The sex of the household head is male or female, recognized as the household head of the unit by members of the household. The wealth index, a measure of household socioeconomic status, was classified in this study as poor (poorest and poorest), middle, and rich (rich and richest). The number of household members in the household is classified as < = 4, 5–6, and 7 + members. The quality of antenatal care was determined by six antenatal care components deemed essential for every pregnant woman (whether the woman’s weight, blood pressure, urine sample, blood sample, information about possible pregnancy-related complications, and iron tablets were provided during an antenatal visit) [42]. In the current study, this was coded as ‘high quality’ or ‘low quality,‘ with high quality referring to a woman who received all six components of ANC and low quality referring to a woman who did not receive all six components of ANC.

### Community-level variables

The community-level variables were created by aggregating individual-level characteristics at the community (enumerated area) EA level, and aggregate variables were classified as high or low based on the distribution of the proportion values calculated for each EA. Four community-level variables are included in this study: community mean distance to the health facility, community socioeconomic status, community ethnic diversity, and community media saturation.

The community’s average distance to the nearest health facility in the EA was referred to as the mean distance to the health facility. A woman is considered accessible to a health facility if she lives in a household within a 5 km radius of the nearest health facility; otherwise, she is inaccessible. The distance to the nearest health facility was calculated using ArcGIS software on 685 EAs whose coordinates were found to be valid [59].

The proportion of poor women in the EAs was used to assess the socioeconomic status of the community. The variable was built using the household wealth index’s poorer and poorest quintiles. The median was used as a cut-off point because the aggregated values are not normally distributed. The proportions less than 50% were coded “0” low, and those greater than 50% were coded “1” high [60].

The number of ethnic groups and their proportional representation in the EA is defined as the community ethnicity diversity index. The score ranges from 0 to 1, with higher scores indicating greater diversity. For ease of interpretation, the score is multiplied by 100; if the majority of an EA population is from a single ethnic group, the EA has a diversity index 0. If the population is evenly distributed among the various ethnic groups, EA diversity reaches 100 [60].

The proportion of women exposed to at least one media (radio, newspaper, television) in an Enumerated Area was used to calculate community media saturation (EAs). The median value was used as a cut-off point because the aggregated community media saturation variable did not follow a normal distribution. The proportion less than 50% was classified as low, while the proportion greater than 50% was classified as high [7].

### Data analysis

The women’s records dataset was used in this study to analyze secondary data from the UDHS 2016. The data was analyzed using STATA version 14.2. Descriptive statistics, presented in percentages, were used to summarize key variables in the univariate data. At the bivariate level, frequencies and cross-tabulations were used to determine how individual and community-level characteristics spread out the outcome variables. Pearson’s chi-square was used to test the relationship between individual and community-level characteristics. The overall categorical variables with p-values of 0.25 in the bivariate model were tested for multicollinearity using the Variance Inflation Factor (VIF). The threshold for VIF was set at 10; any variable with VIF greater than 10 was dropped out of the model. The results show that multicollinearity is absent (mean VIF = 1.28, min VIF = 1.01, max VIF = 1.98), and these variables were fitted to the final multivariate model. The outcome variable was dichotomous, and a multilevel binary logistic regression model was used to test the relationship between individual and contextual variables and the utilization of adequate antenatal care services. To assess individual and community variations, the fixed and random effects were calculated [61] and fitted into multilevel logistic regression model:

$${log}\left[\frac{{\pi }_{ij}}{1-{\pi }_{ij}}\right]{=\beta }_{0}+{\beta }_{1}{x}_{1ij}+{\beta }_{1}{x}_{2ij}+\dots +{\beta }_{n}{x}_{nij}+ uoj+ eij$$

Where: *π*_*ij*_ is the probability of i^th^ individual in the j^th^ community utilizing adequate antenatal care services. *(1-π*_*ij*_*)* is the probability of i^th^ individual in the j^th^ community not utilizing adequate antenatal care services, β_0_ is the log odds of the intercept, β_1_, … β_n_ are the effect sizes of individual and community-level factors, X_1ij_… X_nij_ are independent variables of individual-level and community-level, u_Oj_ the quantities of random errors at community levels and $e$_*ij…*_is the random error at the individual level. Four multilevel binary logistic regression models are employed to test the association between individual and contextual variables and the utilization of adequate antenatal care services. In the initial, empty model (Model 1), no covariate was included. The model examines the random influence of the variability between EAs. The inter-class correlation coefficient (ICC) was calculated to see if the multilevel analysis method is justified by demonstrating the level of variance between EAs.The second models (Model 2) determined the effects of individual-level characteristics on women’s utilization of adequate antenatal care services. The ICC was calculated and observed if there was any change in between-EA variability upon adding the individual-level characteristics to the empty model. The third model (Model 3) introduced community-level characteristics and excluded individual-level characteristics. In the fourth model (Model 4), which is the combined model, both the individual-level and community-level characteristics were fitted to show their net fixed and random effects. The random effect was explained using the inter-Class Correlation (ICC) using the following formula [ICC = σu^2^ / (σu^2^ + π^2^ /3)]. The fixed-effect sizes of individual-level and community-level factors on utilization of adequate antenatal care services were stated using the Adjusted Odds Ratio (AOR) 95% confidence iinterval. The statistical significance level was declared at a p-value less than 0.05 [61]. The “svyset” module in the STATA software was used to account for the complex sample by taking into account the three pieces of design elements; weights, EAs, and strata. The framework for the approximate-level weight of multilevel models was adopted. This method helps correct the inflated type one error and large confidence intervals [58]. The log-likelihood ratio was used to test how adequate the model is, and Akaike Information Criteria (AIC) was used to assess how well the different models fit the data [62].

## Results

### Background characteristics of respondents

Table 1 presents the sociodemographic and other selected background characteristics of women. About one in eight respondents (13.7%) were adolescents (≤ 19 years old), whereas. 64.3% were married, and 61.3% were living together. Close to three-quarters (73.0%), of respondents belonged to male-headed households. Nearly half of the respondents (46.0%) had four or more children. 36% of the respondents belonged to households with over seven members. 60% of the respondents had a primary education level, whereas 76% resided in rural areas. 41% of respondents belonged to the poor bracket of the wealth index. Over a quarter (31.1%) of the respondents belonged to Anglican; meanwhile, 39.5% of them belonged to Catholic denominations. Over three-quarters (79.0%) of the respondents were employed, whereas 73.0% of the women received low-quality ANC content. About 59.0% of the respondents resided in communities that had been deemed not accessible to health facilities, whereas 50.4% of the respondents belonged to communities of low socioeconomic status category. 80% of respondents belonged to less diverse communities. Lastly, 50.4% of the respondents belonged to communities that had low media saturation.

**Table 1:** Percentage distribution of respondents by background characteristics (shown in brackets), and percentage distribution of women by number of ANC visits and background characteristics

Variables	Number(weighted) N, % N = 9416 (100%)	< 4 Times ANC visits (Weighted %)	>=4 Times ANC visits (Weighted %)	*P-*value
**INDIVIDUAL VARIABLES**				
**Age at last birth**				0.000
<=19	1286 (13.7)	39.4	60.7	
20–29	5004 (53.7)	36.4	63.6	
30–39	2628 (27.4)	41.1	58.9	
40+	498 (5.3)	46.4	53.6	
**Marital Status**				0.101
Single	532 (5.7)	37.4	62.6	
Married	4019 (40.6)	37.6	62.4	
Living together	3730 (41.0)	38.7	61.3	
Previous married	1135 (12.8)	42.2	57.8	
**Household Head**				0.795
Male	6890 (73.2)	38.5	61.5	
Female	2526 (26.8)	38.9	61.1	
**Parity**				0.000
1	1852 (20.2)	35.5	64.5	
2–3	3160(34.2)	35.9	64.1	
4+	4404 (45.6)	42.0	58.0	
**Family Size**				0.000
<=4	3066 (33.6)	35.3	64.7	
5–6	2925 (30.9)	39.1	60.9	
7+	3425 (35.6)	41.3	58.7	
**Level of Education**				0.000
No education	1177 (10.4)	44.9	55.1	
Primary	5753 (59.9)	41.0	59.1	
Secondary or Higher	2486 (29.8)	31.7	68.3	
**Place of Residence**				0.000
Urban	1950 (23.3)	33.1	66.9	
Rural	7466 (76.7)	40.3	59.7	
**Wealth Index**				0.000
Poor	4410 (41.4)	43.0	56.9	
Middle	1749 (19.0)	40.0	60.0	
Rich	3257 (39.7)	33.3	66.7	
**Religion**				0.167
Anglican	2977 (31.2)	38.4	61.6	
Catholic	3920 (39.6)	39.2	60.9	
Muslim	1077 (13.7)	35.4	64.6	
Other’s	1442 (15.5)	40.4	59.6	
**Employment Status**				0.019
Unemployed	1870 (21.0)	41.3	58.7	
Employed	7546 (79.0)	37.9	62.1	
**Quality of ANC content**				0.000
Low	6971(73.3)	42.4	57.6	
High	2445 (26.7)	28.3	71.7	
**COMMUNITY VARIABLES**				
**Community Distance to Health Facility**				0.049
Inaccessible	6518 (58.6)	41.9	58.1	
Accessible	2756 (41.4)	33.7	66.3	
**Community Socioeconomic Status**				0.000
Low	5398 (50.4)	42.0	58.0	
High	4018 (49.6)	35.2	64.8	
**Community Ethnicity Diversity Index**				0.028
Less Diverse	7445 (80.2)	38.0	62.0	
More Diverse	1971 (19.8)	39.5	60.5	
**Community Media Saturation**				0.002
Less Saturated	5229 (50.4)	41.3	58.7	
More Saturated	4187 (49.6)	35.9	64.1	
**Total**	**9416**	**38.6**	**61.4**	

### Prevalence of adequate utilization of ANC services across explanatory variables

Table 1 further shows the percentage distribution of women by number of ANC visits and background characteristics. About 5780 (61.4%) of respondents utilized ANC services adequately. Among the individual-level variables examined, the percentage of women who adequately utilized ANC services differed significantly by age, parity, household family size, level of education, residence, household wealth index, and employment status. There is a higher prevalence of adequate utilization of antenatal care services among women aged 20–29 (63.6%) as compared to those aged ≤ 19 (60.7%). The proportion is lower for women aged 30–39 and those aged 40 and above (58.9% and 53.6%, respectively). Only 58.0% of the women of higher (≥ 4) utilized ANC services adequately compared to women of lower parity (1 and 2–3(64.5% and 64.1% respectively)). Similarly, 58.7% of women who belonged to households with large family sizes of at least 7 members utilized ANC adequately compared to those who belonged to households with family sizes of at most 6 ( < = 4 members and 5–6 members (64.7% and 60.9% respectively)). Adequate utilization of ANC services decreased with the level of education women had, women with secondary or higher education (68.3%) utilized ANC services adequately compared to those with primary education and those with no education (59.1% and 55.1% respectively). Adequate utilization is higher among women who resided in urban areas (66.9%) as compared to 59.7% of those in rural areas. Adequate utilization of ANC services also decreased with a decrease in household wealth. The percentage was highest among women who belonged to the rich wealth index bracket (66.7%) compared to those who belonged to the medium and poor household wealth index (60.0% and 56.9% correspondingly). A slightly higher percentage of women who reported being employed (62.1%) utilized ANC services adequately compared to those who were unemployed (58.7%). Lastly, women who received high-quality ANC content utilized ANC services adequately (71.7%) compared to those who received low-quality ANC content (57.6%). All these individual-level variables were statistically significant (p < 0.05).

Bivariate analysis of adequate utilization of ANC services was also examined among community-level variables. The proportion of women who utilized ANC services adequately differed significantly for all community-level characteristics examined. A higher proportion of women residing in communities that were accessible to health facilities (66.3%) utilized ANC services adequately compared to those with limited access to health facilities (58.1%). A higher proportion of women who belong to communities with a high socioeconomic status utilized ANC services adequately (64.8%) compared to women residing in communities with a low socioeconomic status (58.0%). Furthermore, adequate utilization of ANC services was observed among women who resided in less ethnically diverse communities (62.0%) compared to those from diverse communities (60.6%). Lastly, a higher proportion of women who resided in communities with more saturated media (64.1%) utilized ANC services adequately compared to their counterparts with less media saturation (58.8%). All the community-level variables were statistically significant (p < 0.05).

### Determinants of adequate utilization of antenatal care services

#### Results of the random effects

A total of four models were used, with model 1 being an empty model. Model 2 introduced only community-level factors, whereas Model 3 introduced individual-level variables. The final model (model 4) introduced both individual and community-level factors. Results of the null model (model 1) show a statistically significant variation in the odds of adequate utilization of antenatal care services with a community-level variance of 46.0%. The ICC in the null model suggested that 12.8% of the total variability in the adequate utilization of antenatal care services was attributed to the differences between communities. In the full model, which is adjusted for the individual and community-level factors, the community variance was reduced to 45.0% and remained statistically significant. A total variance of 12.5% of adequate utilization of antenatal care services can be attributed to community-level characteristics (see Table 2).

**Table 2:** Multilevel Analysis of Odd Ratios on the Effect of Individual and Community Level factors influencing adequacy utilization of ANC among women in Uganda, UDHS 2016

Variables	Model 1	Model 2	Model 3	Model 4
	Empty Model	Individual-level	Community-level	Individual/ Community-level
	COR (95% CI)	COR (95% CI)	COR (95% CI)	AOR (95% CI)
**INDIVIDUAL VARIABLES**				
**Age at last birth**				
<=19		1.00		1.00
20–29		1.03 (0. 85-1.24)		1.03 (0.85–1.24)
30–39		0.91(0.73–1.14)		0.91 (0.73–1.14)
40+		0.81(0.59–1.11)		0.81 (0.59–1.11)
**Marital Status**				
Single		1.00		1.00
Married		1.18 (0.91–1.52)		1.18 (0.92–1.52)
Living together		1.07 (0.85–1.37)		1.08 (0.85–1.36)
Previous married		0.94(0.71–1.23)		0.94 (0.71–1.23)
**Parity**				
1		1		1.00
2–3		0.95 (0.80–1.13)		0.95 (0.80–1.12)
4+		0.78 (0.73–1.08)		0.74 (0.72–1.07)
**Family Size**				
<=4		1.00		1.00
5–6		0. 95 (0.80–1.13)		0.92 (0.80–1.06)
7+		0.88 (0.73–1.08)		0.92 (0.79–1.06)
**Level of Education**				
No education		1.00		1.00
Primary		1.11 (0.93–1.33)		1.11(1.07–1.63)
Secondary or Higher		1.32** (1.07–1.63)		1.32**(1.07–1.63)
**Place of Residence**				
Urban		1.00		1.00
Rural		0.95 (0.79–1.13)		0.98 (0.80–1.20)
**Wealth Index**				
Poor		1.00		1.00
Middle		1.08 (0.92–1.26)		1.07 (0.92–1.26)
Rich		1.29** (1.11–1.49)		1.27**(1.09–1.49)
**Religion**				
Anglican		1.00		1.00
Catholic		0.96 (0.83–1.09)		0.96 (0.84–1.10)
Muslim		0.96 (0.76–1.19)		0.97 (0.78–1.19)
Other’s		0.90 (0.76–1.08)		0.91 (0.76–1.08)
**Employment Status**				
Unemployed		1.00		1.00
Employed		1.25** (1.10–1.44)		1.26** (1.10–1.44)
**Quality of content of ANC**				
Low		1.00		1.00
High		1.79***(1.58–2.02)		1.78***(1.58–2.02)
**COMMUNITY VARIABLES**				
**Community Distance to health facility**				
Not Accessible			1.00	
Accessible			1.00 (0.88–1.15)	
**Community Socioeconomic status**				
Advantaged			1.00	1.00
Disadvantaged			1.38***(1.17–1.61)	1.09 (0.89–1.33)
**Community Ethnicity Diversity Index**				
Less Diverse			1.00	1.00
More Diverse			0.82*(0.70–0.96)	0.85*(0.73–0.99)
**Community Media Saturation**				
Less Saturated			1.00	1.00
Saturated			1.11 (0.95–1.30)	0.99(0.84–1.16)
Random effect results:				
PSU Variance (95% CI)	0. 46 (0.38- 0.56)	0.45(0.37–0.54)	0.44(0.36–0.53)	0.45(0.37–0.54)
ICC	12.78%	12.53%	12.29%	12.53%
Wild chi-square and p-value	Ref	χ^2^ = 190.28, p < 0.000	χ^2^ = 26.53, p < 0.000	χ^2^ = 197.88, p < 0.000
Model fitness:				
Log-likelihood	-2285933.2	-2242600.7	-2237368.2	-2241196.3
AIC	4,502,698	4,417,975	4,460,695	4,388,384
PSU	649	649	649	649
N	9,416	9,416	9,274	9,416

Note: The Empty Model contains no variables, but it partitioned the variance into two component parts. AIC = Akaike Information Criterion, BIC = Bayesian Information Criterion, CI = Confidence Interval, ® Reference category, AOR = Adjusted Odd Ratio, COR = Crude Odd Ratio, Significant level, ***p = < 0.001, **p < 0.01, *p < 0.05

#### Results of the fixed effects

The model fit for the four models was examined, and the one with the smallest AIC was chosen as the best fit for the data. Based on Model 4, which had the lowest AIC statistic, the fixed effects results were determined. This model was adjusted for individual and community-level factors with a small AIC compared to the other models, indicating its strong fit with the data. In the multilevel analysis, women’s level of education, wealth index, employment status, quality of ANC content, and community ethnicity diversity index showed statistically significant associations with the adequate utilization of antenatal care services among women in Uganda at a 5.0% level of significance. The model indicates that women with secondary or higher education were 32.0% (AOR = 1.32, 95% CI; 1.07–1.63) more likely to adequately utilize antenatal care services compared to those with only primary or no education. Women who belong to the rich category of the household wealth index bracket were 27.0% (AOR = 1.27, 95% CI; 1.09–1.49) more likely to utilize adequate antenatal care than those who were poor. Employed women were 26.0% (AOR = 1.26, 95% CI; 1.10–1.44) more likely to utilize ANC services adequately compared to those who were unemployed. Women who received high-quality content for antenatal care services were 78.0% (AOR = 1.78, 95% CI; 1.58–2.02) more likely to utilize adequate antenatal care services compared to those who received low-quality ANC content. Moreover, at the community level, women living in ethnically diverse communities were 15.0% (AOR, 0.85, 95%CI; 0.73–0.99) less likely to adequately utilize ANC services compared to their counterparts (see Table 2).

## Discussion

This study examined the effects of individual and community-level factors associated with adequate utilization of antenatal care services among women in Uganda. Overall, the prevalence of adequate utilization of antenatal care services in Uganda was 61.4%, and this finding corroborated a study from Uganda [49]. Comparatively, this proportion was lower than that of Congo (73.4%), Cameroon (62.6), Gabon (68.4%), Gambia (78.1%), and Ghana (86.0%) Lesotho (74.2%), Liberia (73.0%), Namibia (63.3%), South Africa (78.1%), Zambia (64.3%) and Zimbabwe (76.2%) [35]. This study identified several individual and community-level factors associated with adequate utilization of antenatal care services. More specifically, the results showed that women’s level of education, wealth index, employment status, and quality of content for antenatal care were among the important predictors of individual-level factors. Moreover, at the community level, the community ethnic diversity index was the influencing factor.

Women’s level of education was associated with adequate utilization of antenatal care services in Uganda. As expected, the study found that women who had secondary or higher education were more likely to adequately utilize antenatal care services compared to those with primary or no education. This finding corroborates other studies from sub-Saharan Africa [37, 63]. Generally, education empowers women with a better knowledge to process information regarding the use of maternal healthcare services, and such women are in a better position to make decisions regarding their health in households [44, 49]. Therefore, this implies that women with primary or no education are at higher risk of underutilizing antenatal care services. There is a need to increase maternal healthcare education among women with primary or no education.

As expected, women in the rich bracket of the wealth index were more likely to adequately utilize antenatal care services than those in the poor bracket. This finding is consistent with other studies in developing countries and sub-Saharan Africa that found an association between the wealth index and adequate utilization of antenatal care services [38, 64]. Similarly, we found that women who were employed were more likely to adequately utilize antenatal care services compared to those who were not employed. These findings are consistent with other studies from Ethiopia and Uganda [33, 45]. Working status among women was associated with income and a level of autonomy at the household level. This is because women with higher socioeconomic classes have a greater ability to pay for both direct and indirect healthcare costs [65]. Furthermore, travel and service costs were shown to be the primary deterrents to receiving ANC in low and middle-income countries by Simkhada et al. [41]. However, a meta-analysis has also shown that, even if ANC services and transport are free, low-income women may still be unable to use them because they are too expensive to utilize during their busy days caring for their families and homes [42]. Policies aimed at empowering women in Uganda through income-generating activities could go a long way in ameliorating poverty-stricken women, especially in rural and urban slums [49].

Furthermore, women who reported receiving high-quality antenatal care content were more likely to have utilized adequate antenatal care services than their counterparts. The findings of this study are consistent with other studies that have established a positive relationship between receiving high-quality content of ANC services and adequate utilization of antenatal care services [39, 40]. Women who attended ANC services at least four times based on the WHO-focused ANC framework are more likely to receive all items of ANC content [40]. Conversely, the provision of recommended ANC packages across providers would increase the number of visits women make, leading to better maternal and foetal outcomes. Therefore, mothers must be educated on the need for regular visits to antenatal care services, which will lead to them receiving high-quality content of antenatal care services from skilled health personnel [40].

Finally, women from ethnically diverse communities were less likely to utilize antenatal care services adequately compared to their counterparts. Similarly, research in Uganda and elsewhere has shown that high ethnic diversity is associated with poor health outcomes [66]. However, a study by Ononokpono et al. [43] found that women who reside in more ethnically diverse communities are associated with high-level social networks, which could encourage improved information sharing about maternal healthcare services and their utilization [43]. The results undoubtedly demonstrate how complex and even difficult it is to understand the effects of ethnic diversity on outcomes for mother and child health [54].

### Strengths and limitations of the study

This study used nationally representative data, and the results obtained can be generalized to the entire nation. The use of multilevel-level analysis in this study is a powerful tool for which limited studies have been conducted to date. The use of quality of antenatal care as an explanatory variable in the context of Uganda is one of its first kind. Most studies used it as an outcome variable, not a predictor variable. The other major contribution of this study is the use of level-weights proposed by Elkasabi et al. [58], which helps to account for its complex nature of DHS dataset. Therefore, the design weights account for unequal selection probabilities and, in due course, help to eliminate biased parameter estimates [57]. Despite its strengths, this study has limitations. This study used a focused antenatal care framework through which the data was collected, and the newly adopted positive pregnancy framework could not be applied because the sample size of women who used ANC services at least eight times was insufficient [3, 4]. Besides, there is the possibility of memory bias due to the retrospective nature of the study, and the missing data may not be verified due to the secondary nature of the data. Other constraints could be related to community-level factors aggregated from individual-level data, which could lead to an ecological error. Above all, these findings provide policy-makers and other stakeholders with reliable information for policy-making and implementation.

## Conclusion

This study found that only 61.4% of the women in Uganda utilized adequate antenatal care services. The study further revealed that women’s level of education, wealth index, employment status, quality of content of ANC services, and community ethnicity diversity predicted the adequate utilization of antenatal care services in Uganda. Due to the less likelihood of uptake of adequate antenatal care services among the poor women, the Ugandan government has to develop poverty alleviation projects targeting the poor women. Furthermore, the Ugandan government, through the Ministry of Health and health workers, must implement maternal education programs aimed at women with primary or no education in order to encourage them to use antenatal care services to the fullest extent possible. Research is required to critically examine the association between ethnicity diversity and the utilization of antenatal care services in Uganda.

## Data Availability

The dataset can be accessed through this website; https://dhsprogram.com/data/dataset_admin/login_main.cfm?CFID=6055127&CFTOKEN=416a39e1e52181a9-CCE2DAA5-A212-565 C-40BD6F8E8C8E5041. Registration is required. This study used the UGIR70FL (Individual Recode –Women with completed interviews – Uganda, 2016).

## Abbreviations

ANC: Antenatal Care
AIC: Akaike Information Criterion
CMC: Century Month Code
CI: confidence Interval
EA: Enumerated Areas
HSDP: Health Sector Development Plan
ICC: inter-class correlation
OR: odd ratio
UDHS: Uganda Demographic and Health DHS Demographic and Health Surveys
AOR: Adjusted odd ratio
WHO: World Health Organization
